# A Treatise on the Diseases of the Chest; Being a Course of Lectures Delivered at the New York Hospital

**Published:** 1852-05

**Authors:** 


### BIBLIOGRAPHICAL NOTICES.

---

*A Treatise on the Diseases of the Chest; being a Course of Lectures delivered at the New York Hospital.* By JOHN A. SWETT, M.D., Physician to the New York Hospital; Member of the New York Pathological Society. New York, D. Appleton & Co., 1852. 8vo. pp. 585.

This work, as we are informed by the author, was originally published in the New York Lancet some ten years since. At "the repeated solicitation of his medical friends," he has revised these lectures, and has added those new facts which an enlarged experience, both in private practice and in the New York hospitals, has given to him. The form of lectures is, however, retained, and therefore the work to a certain extent loses in value as a regular treatise upon the diseases of the chest; for we do not think that the form of phraseology which may be admirably adapted to a lecture is nearly so well fitted for a thorough description of the phenomena and modes of treatment of disease. This objection is, however, of very little moment; we are disposed to allow to authors the greatest possible latitude in selecting the mode of treating the subject, which may suit their own fancy; and whether they choose to divide their work into chapters or cut it up into lectures, matters but little.

After the first two introductory lectures in connection with the physical exploration of the chest and with the rational symptoms of its diseases, the author proceeds at once into his subject, and takes up the history of bronchitis. In one of these preliminary lectures, he speaks of pain, dyspnœa, cough and expectoration as the four rational symptoms of diseased lung. Pain, Dr. Swett thinks, with great justice, is much more marked in pleurisy than in any other disease of the chest. We agree with him that it is almost the only disease in which the pain is violent, but not that the pain is more severe in slight cases than in those of more gravity. Pain of course becomes slight when there is a large effusion into the chest; but in almost all cases of very severe pleurisy, whether complicated with pneumonia or not, the pain is always great as long as there is little effusion into the chest; afterwards it is much lessened.

After passing through the subject of bronchitis, the author proceeds to enter upon the history of pneumonia. He remarks that chronic bronchitis, (in which we agree with him,) is not generally much under the influence of medicine. Still it is all-important to decide that a case of chronic cough depends upon bronchitis, and not upon any more dangerous disease of the lungs with which it may be readily confounded.

The description of the physical alterations of pneumonia might, we think, be much more full and more exact than they appear in Dr. Swett's work. Thus he speaks of abscesses following pneumonia as rare. They are, it is true, very rare after death, but we have good reason to believe that pneumonia may now and then end in abscess; although when this result takes place, the disease will almost always terminate favorably. Abscesses in the lung, our author states, are favored by two circumstances—one is the effusion of pus into the cavity of the pleura. "When this has occurred, it will sometimes happen that a distinct abscess will form in the lung, which may finally open into a bronchus, or communicate with the pleura." We have certainly never witnessed a case of the kind, and we may conclude that it must be at least very rare. The metastatic abscess is scarcely to be classed as one of the terminations of inflammation; it is simply a cause of it, not an effect; with this view we may infer that Dr. Swett also agrees.

Gangrene of the lungs, Dr. Swett states, is usually classed as an effect of inflammation. We were scarcely aware of that. For many years gangrene has been regarded as very rarely connected with pneumonia; sometimes, however, it is in part dependant upon it, and may follow an inflammation of the lungs, just as it may upon one of any other part of the body. In many cases of the disease, gangrene of the lungs begins at once without being preceded by any obvious inflammatory action in the part.

In speaking of the treatment of pneumonia, the author has given us the statistics of M. Grisolle on the effects of treatment. These results coincide very nearly with what is generally believed. Bloodletting is the most important remedy, diminishing the duration of mild cases, and lessening the mortality in those of a severe class; but after bleeding, the author considers the most powerful remedy to be antimony. Eight to ten grains of it he considers as the medium dose during the twenty-four hours. Now, from our own experience, after having used antimony very faithfully and carefully for many years, we are led to a very different conclusion. It is a powerful remedy, perhaps the most powerful; but, at the same time, when used in the large doses recommended, is by no means a safe one; we have seen extensive ulcerations of the bowels produced by its use, even in cases in which no diarrhœa followed it, so that on the whole we would administer antimony very rarely in large doses as a contra-stimulant. Mercury is certainly about as good a remedy in treating pneumonia as antimony, and is free from many inconveniences which belong to the use of the latter medicine—we mean when employed as a contra-stimulant; but there is no objection to the use of antimony as a sedative expectorant when given in small doses.

Pleurisy our author also treats with some detail, and of course he enters into the pathological changes and mode of treatment. Then after passing through emphysema of the lungs and some other affections, he passes to the subject of tubercles in the lungs. The pathological changes in the lungs are of course described at length, although less carefully than they might be; but the tuberculous diseases of other organs, which form so large and important a part of the history of these diseases, are almost passed over. It seems to us that in writing the history of pulmonary phthisis, it is all-important to keep in view the fact that the deposition of tubercles and the changes produced by them constitute the anatomical characters of the disease. It is true these bodies are commonly more hurtful in the lungs than in other organs, but the lungs are far from being their exclusive seat of origin; hence it is most important to keep in view the fact that consumption is a general tuberculous disease specially determined towards the lungs.

The history of the causes of tubercles in the lungs is treated of in much detail. The usual circumstances that favor the development of tubercles are stated, as well as some of the means of resisting their attack. Amongst the antagonising causes which check the development of phthisis, the author mentions one, of the truth of which we have long been abundantly convinced—that is, that the intemperate use of ardent spirits to a certain extent diminishes the chances of an attack of consumption. Of the two modes of death—by drunkenness or consumption—we trust that no one would have a moment's hesitation in preferring the latter to the former. Still the question arises, whether a physician may, without serious mischief to the patient, order the habitual use of wines or spirits in cases in which, from the history of the family of the individual, as well as the actual circumstances of the case, an invasion of pulmonary consumption seems to be imminent. As a rule, we would object to any medical opinion which might give to a patient the idea that alcohol is the best preservative against consumption, but in cases in which confidence may be reposed in the self-control of the individual, we believe that very often the physician is amply justified in recommending the use of moderate quantities of alcoholic drinks. Still, every caution must be used in giving advice of the kind, or the remedy may prove much worse than the disease. When consumption is once developed, however, the free use of alcoholic drinks is almost always injurious, often greatly increasing the fever. Under these circumstances they should be given only in small quantities as a prescription for the disease. But there are cases in which, from the emaciated aspect of the patient, and from a hereditary tendency to consumption, the onset of this disease is with reason feared, in which the moderate, careful use of stimulating drinks may with propriety be advised by physicians, who believe that their duty is to save life, and who would administer stimulating drinks on the same principle that they would give cod-liver oil or any other strictly medicinal remedy.

A number of remedies are afterwards considered by the author, in the treatment of phthisis. Cod-liver oil he is disposed to distrust, and thinks it is soon to be forgotten. With this opinion we are far from coinciding. We believe that some oily substances have long been recognized as very useful in the treatment of phthisis, and that cod-liver oil answers the indications which demonstrate the utility of these oleaginous remedies better than any other material of the kind. There is no doubt that many individuals laboring under decided tuberculous disease are vastly improved by a course of cod-liver oil; the disease is apparently arrested, or, as many physicians say, is cured. We must confess, however, that we have been stupid enough to see very little proof of positive actual cure; but after a time the disease again becomes more active, and will resume its march until the termination. Still the remedy, on the whole, is by far the best yet discovered against phthisis, and we cannot but think Dr. Swett has treated it much too cavalierly. His remarks as to the use of the other remedies commonly used in the treatment are generally judicious; as to opium he lays down some well-timed cautions.

Dr. Swett next devotes a chapter to cancer of the lungs. This disease in the United States is certainly very rare, and then it occurs almost always in connection with some external cancerous disorder.

On the whole, although the work of our author, as far as regards the diseases of the air passages, is by no means free from faults of arrangement and some errors of more importance, it is in the main sound and based upon data which are received as facts in the best European schools. These are corroborated by his own experience, which, from the position he has enjoyed at the New York hospital, has been very extensive. We are but little disposed to criticise the arrangement of his subject, for that in part depends upon the radical defect we have already alluded to—that is, the arrangement of the work in lectures; but in part it may be ascribed to some errors as to the relative importance of particular subjects.

After having passed through the diseases of the lungs, the author then proceeds to the diseases of the heart. This part of the work is much more carefully executed than that devoted to the lungs. We do not know if it is a part which was originally published in the New York Lancet or not; if it is, it has certainly been attentively revised. After passing through a careful statement of the sounds of the heart in health, the author proceeds to an enumeration of the signs and symptoms of a diseased heart. This part of the subject is well treated of, and is quite thoroughly detailed. The different facts attested by Bizot, Hope, and Pennock are mentioned in connection with the portions of the text that belong to them. Passing to individual diseases of the heart, the author commences with pericarditis. After going through the history of this disease, he next proceeds to its causes. Amongst these he classes inflammatory rheumatism as the most powerful. We must confess that we are not a little disposed to doubt this. Pericarditis certainly does often occur conjoined with rheumatism, but less frequently than endocarditis; and we are induced to think that it occurs at least as often with pneumonia or pleurisy as with rheumatism. We agree with the statements given by some of the authors to whom Dr. Swett refers as to the comparative rarity of pericarditis in rheumatism.

Endocarditis is rather singularly mixed up in the arrangement of our author with pericarditis—that is, he enters upon the treatment of pericarditis after commencing the subject of endocarditis. The author remarks very truly that "the great cause of endocarditis is acute articular rheumatism;" and he then proceeds to say, that "the remarks that I made in relation to this cause, when speaking of pericarditis, apply precisely to endocarditis." We should say they do, with the very important proviso that endocarditis is rarely absent in a case of acute articular rheumatism, while pericarditis is present with almost equal rarity; that is, the one occurs as the rule, and the other the exception. It is indeed difficult to say why pericarditis should be more frequently present than pleurisy in this disease, the pericardium and pleura bearing always an exceedingly close relation together in their morbid relations; while the same close connections certainly do not exist between the lining membrane of the heart and the pericardium.

The author then states that "the rational and the constitutional symptoms of endocarditis resemble so nearly those of pericarditis." This is to a certain extent true, and increases somewhat the difficulty of diagnosis. Although we regard decided pain as rather indicative of pericarditis, still in some cases of endocarditis there is pain, but it is usually slight and not constant, although it is indicative of obstruction to the circulation. No one, therefore, without the aid of other signs can rely upon the general symptoms, although these are tolerably good guides for the diagnosis. The most certain guide, as our author states, is the blowing sound. This is similar in some respects to that developed in anemia, although a certain degree of experience would point out the distinction between these sounds, which although nearly similar, are not precisely identical; the bellows sound resulting from anemia forming a peculiar musical tone very different from that of endocarditis. It is, however, much more easy to point out these differences in living patients, than to render them perceptible from a simple description. The author goes on to state that many cases of endocarditis are not marked by severe symptoms, and would be entirely latent if it were not for the bellows sound. In this we agree with him, but we may state that it is always the bellows sound appropriate to endocarditis, which has not the musical tone of that belonging to anemia.

The author afterwards passes on to another cause of confusion which he fancies to exist between two of the sounds of pericarditis and endocarditis. As to this cause of confusion we are obliged to give his own words:

"I cannot help alluding still further to the characters of the two characteristic morbid sounds heard in cases of acute pericarditis and of endocarditis—the friction sound and the blowing sound: for I am well aware, that no subject connected with the science of auscultation, has puzzled its followers more than this. For a long time no accurate distinction was made in the character of these sounds—they were constantly confounded with each other. After a time, however, the true distinction between a blowing sound and a friction sound was established. The ear became accustomed to discriminate them by the fundamental character of each—the kind of sound. Other circumstances, already alluded to, were noticed in connection with each. The superficial, diffused and moveable nature of the friction sound, as well as its comparatively short duration, and its being usually double, were carefully noted. While the blowing sound, by its greater concentration and fixedness, its tendency to pass along the course of the great vessels, or to exist at the apex, by its being usually single and continuing for a long period, became impressed with marks which, although not perfectly characteristic, are yet often of great value in doubtful cases. If you could readily distinguish a blowing sound from a friction sound in all cases, that would be enough for the diagnosis. But you cannot. A

friction sound may hardly be distinguished from a soft blowing sound, and both may become equally harsh and grating."

Now it certainly seems to us that the author has imagined that those sounds are very similar, or at any rate not unlike. This is undoubtedly an error. The friction sound is evidently external to the heart, and synchronous with the movement of the whole organ against the walls of the chest. The bellows sound is very different, and strictly coincident, generally with the systole, sometimes with the diastole of the heart.

Bloodletting and mercury are of course mentioned as the most efficient agents in checking inflammation of the serous membrane of the heart. They are such, undoubtedly, but they are not always required or even always admissible; if the patient be taken with these inflammations when he is already much exhausted, then general bleeding is of course entirely out of the question. Yet these very cases sometimes get well with almost as much rapidity as those in which the proper treatment has been called for during the course of the disease.

Dr. Swett next proceeds to the subject of the enlargement of the heart. His remarks on this subject are in general just and appropriate, especially when he alludes to the irregularity of the pulse. He remarks with great correctness that this is far from being a sign of disease; in many cases it is certainly unconnected with any organic affection.

Valvular diseases of the heart of course are of no little importance. We regret that the limits of this notice will prevent us from examining the diagnostic characters given by our author. He next passes through other affections of the heart of less gravity, and finally terminates the course of lectures with aneurism of the aorta.

We may, in concluding our remarks, state, that although we have found the work in several respects imperfect, and occasionally very different from what we have considered as strictly accurate in the details, yet on the whole it shows that the author is well acquainted with his subject, and in most respects it is an accurate and well digested compend of the state of our knowledge upon the diseases of the chest. A little more condensation of certain portions of it would, however, have left room for the author to have written more at large upon some points over which he has passed very lightly.

